# The Athlete's Brain: Cross-Sectional Evidence for Neural Efficiency during Cycling Exercise

**DOI:** 10.1155/2016/4583674

**Published:** 2015-12-27

**Authors:** Sebastian Ludyga, Thomas Gronwald, Kuno Hottenrott

**Affiliations:** ^1^Department of Sport, Exercise and Health, University of Basel, Gellertstrasse 156, 4052 Basel, Switzerland; ^2^Institute of Performance Diagnostics and Health Promotion, Martin-Luther-University Halle-Wittenberg, Weinbergweg 23, 06120 Halle (Saale), Germany; ^3^Institute of Sport Science, Exercise and Health, Otto-von-Guericke-University Magdeburg, Zschokkestraße 32, 39104 Magdeburg, Germany; ^4^Department of Sport Sciences, Martin-Luther-University Halle-Wittenberg, Von-Seckendorff-Platz 2, 06120 Halle (Saale), Germany

## Abstract

The “neural efficiency” hypothesis suggests that experts are characterized by a more efficient cortical function in cognitive tests. Although this hypothesis has been extended to a variety of movement-related tasks within the last years, it is unclear whether or not neural efficiency is present in cyclists performing endurance exercise. Therefore, this study examined brain cortical activity at rest and during exercise between cyclists of higher (HIGH; *n* = 14; 55.6 ± 2.8 mL/min/kg) and lower (LOW; *n* = 15; 46.4 ± 4.1 mL/min/kg) maximal oxygen consumption (VO_2MAX_). Male and female participants performed a graded exercise test with spirometry to assess VO_2MAX_. After 3 to 5 days, EEG was recorded at rest with eyes closed and during cycling at the individual anaerobic threshold over a 30 min period. Possible differences in alpha/beta ratio as well as alpha and beta power were investigated at frontal, central, and parietal sites. The statistical analysis revealed significant differences between groups (*F* = 12.04; *p* = 0.002), as the alpha/beta ratio was increased in HIGH compared to LOW in both the resting state (*p* ≤ 0.018) and the exercise condition (*p* ≤ 0.025). The present results indicate enhanced neural efficiency in subjects with high VO_2MAX_, possibly due to the inhibition of task-irrelevant cognitive processes.

## 1. Introduction

Neural plasticity is the brain's ability to change in response to normal developmental processes, experience, and injury. The mechanisms involved in plasticity in the nervous system are thought to support cognition, meaning that brain function is improved. There is evidence from neuroimaging that subjects achieving high scores on cognitive tasks compared to low performers complete these tests with lower cortical activation particularly in the frontal brain region [1, 2]. This observation has led to the “neural efficiency” hypothesis, assuming that experts with high cognitive performance are characterized by a more efficient cortical function [3]. Basten et al. [2] suggest that this particular characteristic can only be inferred from brain activation, when experts and nonexperts are compared at similar behavioral performance. Within the last years this hypothesis has been extended to experts of movement-related tasks [4]. In this respect, standard EEG techniques have been used to investigate the effect of performance level on the allocation of cortical resources during motor tasks. During the preparation of a shot, Haufler et al. [5] observed less cortical activity in skilled shooters compared to novice subjects. In line with this finding, Del Percio et al. [6] also found decreased cortical activity in experts prior to a shot requiring high precision and interpreted this observation as index for selective event- or task-related cortical activation. Neural efficiency has also been confirmed in professional piano players, who completed a motor task involving finger movements with lower cortical activity than less skilled controls [7]. Although these findings consistently support the efficiency of cortical function in athletes, some evidences from tasks requiring movements in response to visual stimuli question the general applicability of this hypothesis. In this respect, Hung et al. [8] reported higher lateralized readiness potentials during Posner's visuoattentional task in elite tennis players compared to nonathletes. In line with these results, Endo et al. [9] showed that athletes had greater motor cortex activity than nonathletes during a task, in which subjects were asked to abduct the right index finger in response to visual stimuli. Evidence from longitudinal studies investigating effects of motor training consistently supports the efficiency of cortical function in high performers, because motor cortex activity decreased despite improvements on task performance after a training period [5, 10, 11]. Similarly, 4 weeks of endurance training in cyclists led to improved aerobic performance but decreased brain cortical activity during exercise [12]. Based on those findings, the neural efficiency hypothesis might also be applicable to endurance athletes. So far researches have not yet investigated efficacy of cortical function during exercise between subjects of different aerobic performance levels.

However, the current state of research indicates that resting EEG rhythms predict neural efficiency during cognitive and sensorimotor tasks. Alpha synchronization at rest represented by high EEG alpha power is related to increased cognitive and motor performance [13, 14]. In line with these findings, elite karate athletes compared to nonathletes show higher alpha-1 power at parietal and occipital regions at rest with eyes closed [15]. Moreover, training levels also influence the reactivity to eyes opening, so that athletes maintain higher alpha power than nonathletes [16]. Whereas alpha is considered the dominant rhythm in the human brain during mental inactivity, beta activity is regarded as index of information processing at the cognitive level [17]. In previous studies increased alpha power and/or decreased beta power, reflected by a higher alpha/beta ratio, were associated with a decreased level of arousal or vigilance [18, 19]. This pattern is considered normal for the resting state due to a lack of task-specific activation demands. However, endurance exercise increases arousal due to preparedness for external input [20]. Applying the neural efficiency hypothesis, high cortical function despite minimal energy consumption should be indicated by an increased alpha/beta ratio in high performers. In previous trials, high and low performers were differentiated by their individual training levels or participation in competitions [4, 15, 21]. For endurance athletes, maximal oxygen consumption allows a more appropriate and objective classification, because this variable is considered “gold-standard” and predicts time-trial performance [22]. This is due to the concept that an athlete's maximal oxygen consumption is mainly determined by the oxygen delivery to the mitochondria and its utilization, which in turn are limiting the oxidative production of ATP required for the energy supply of the working muscles [23].

As neural efficiency is task-related, it is necessary to study brain cortical activity in athletes directly during endurance exercise. The majority of studies investigating brain function while moving predominantly examined subjects during cycling exercise [12], because it does not create stepping impacts that provoke strong neck muscle contractions and electrode movements [24]. Furthermore, experienced cyclists are thought to be able to maintain a stable body position even at demanding workloads, so that the likeliness of movement-related artefacts in the EEG signal is reduced.

The present study investigates whether or not aerobic power has an effect on the alpha/beta ratio during exercise. Additionally, the authors examine if resting EEG predicts brain cortical activity during exercise. In line with the neural efficiency hypothesis subjects with high maximal oxygen consumption were expected to show less cortical activity during cycling exercise.

## 2. Methods

### 2.1. Participants

Cyclists and triathletes with a minimum cycling training volume of 4 hours per week were directly recruited from local sports clubs. Eligible subjects had to be healthy, between 18 and 35 years, nonsmokers, and right handed. Before the trial commenced all participants underwent health screening following the S1-guideline of the DGSP [25], which included personal anamnesis, orthopaedic assessment, and measurement of resting ECG and blood pressure. Any complications limiting the performance or safety of exercise led to exclusion from the study. Following the screening process, 11 female and 18 male subjects were deemed eligible and provided informed written consent. The participants' characteristics are displayed in Table 1. All study procedures were approved by the local ethics committee and followed the guidelines of the Declaration of Helsinki.

### 2.2. Study Design

The present investigation was designed as cross-sectional trial. For each subject two laboratory visits separated by 3 to 5 days were scheduled. In the first laboratory session aerobic and anaerobic performance were assessed during ergometer cycling. The second visit included recording of EEG rhythms at rest and during endurance exercise at constant workload. Based on maximal oxygen consumption measured on the first visit, the sample was split into a group of lower aerobic power (LOW; VO_2MAX_ ≤ 49 mL/min/kg; *n* = 15) and a group of higher aerobic power (HIGH; VO_2MAX_ ≥ 50 mL/min/kg; *n* = 14). Brain cortical activity was then compared between both groups.

### 2.3. Exercise Testing

For the testing procedures, environmental temperature was held constant at 20°C and nutrition was standardized (last main meal—2 h; 500 mL—30 min). Following the assessment of body weight, subjects completed an incremental test with spirometry (Cortex Medical, Metamax 3b, Germany) on a high performance cycling ergometer (FES, Germany). After a 5 min warm-up at 80 W (f)/100 W (m), the workload was increased by 25 W/3 min until volitional exhaustion. This was defined as the inability to maintain a cadence of at least 60 rpm at the given workload. Heart rate and respiratory parameters were recorded continuously. Using the enzymatic-amperometric method (Dr. Mueller Geraetebau, Super GL Ambulance, Germany), lactate concentration was analysed in blood taken from an ear lobe after each increment. Collected data were processed with WinLactat (Mesics, Germany). According to the Dickhuth model [26], the individual anaerobic threshold (*P*_IANS_) was derived from the lactate-power curve. Additionally, VO_2MAX_ was determined as highest value over a 30 s period.

On the second laboratory visit EEG was recorded under the following conditions: resting with eyes closed while sitting on the ergometer (2 min) and during cycling at constant load. The exercise protocol included a 5 min warm-up (80 W [f]/100 W [m]), 30 min at 100% of the individual anaerobic threshold, and a 5 min cool-down at a low workload (80 W [f]/100 W [m]). Brain cortical activity was recorded over 1 min at 10 and 20 as well as 30 min and subjects maintained a cadence of 90 rpm. Heart rate and cadence were displayed in real-time on a monitor placed in the subjects' natural viewing direction. Prior to the assessments, all subjects were instructed to stabilize their body position and avoid head movements and contractions of the jaw throughout the cycling exercise.

### 2.4. EEG

Using a breathable cap, active AgCL electrodes were placed on the subject's scalp to record EEG from frontal (F3, F4, Fz, F7, and F8), central (C3, C4, and Cz), and parietal (P3, P4, Pz, P7, and P8) brain regions. The electrical reference and ground electrode were located at FCz and AFz, respectively. The electrodes, which were positioned according to the international 10:20 system, were filled with an electrolyte gel to reduce impedance below 10 kΩ. The EEG signal was amplified by the QuickAmp system (BrainVision, Germany) and sampled at 512 Hz. Following the data acquisition, the Analyzer 2.0 (BrainVision, Germany) was used for processing the EEG recordings. After a reduction of the sampling rate to 256 Hz, data were digitally band-pass filtered (time constant 0.0318 s; 24 dB/octave) and a frequency range of 3.0 to 40.0 Hz remained for analysis. The recorded epochs represented the 10th, 20th, and 30th minutes during constant load cycling at *P*_IANS_. A systematic protocol for artefact detection was employed to detect artefacts within those epochs [12]. Following a manual inspection the artefacts tagged by the software were either rejected or confirmed. The remaining artefact-free epochs were divided into several 2 s segments. Subsequently, those segments were averaged individually within each recording period. By using a Hanning window (20%), the EEG data were Fast-Fourier-transformed (FFT) to calculate power values in the alpha (7.5–12.49 Hz) and beta (12.5–32 Hz) frequency domain. Mean activity in both bandwidths was exported. Subsequently, the alpha/beta ratio was calculated and averaged over the epochs representing the recordings during exercise. Accurate test-retest reliability for the alpha and beta power during cycling at 90 rpm has been reported with the EEG acquisition and processing methods used in the present study [27].

### 2.5. Statistics

Statistical analyses were processed using SPSS 22.0 (IBM, USA) for Windows. Prior to group comparisons, Gaussian distribution of the collected data was verified by the Shapiro-Wilk test. One-way ANOVA (factor: group) was used to compare anthropometric measures between medium and high fit subjects. The effect of maximal oxygen consumption on alpha/beta ratio was analysed by applying 2 (group: LOW, HIGH) × 2 (condition: rest, exercise) × 3 (region: frontal, central, and parietal) ANOVA. In a subsequent analysis frequency (alpha, beta) was included as additional factor to assess the effect of maximal oxygen consumption on EEG spectral power. In case of nonsphericity Greenhouse-Geisser correction was applied. Between-subjects and within-subjects effects as well as interactions were reported and further analysed using Fischer's LSD post hoc test. Subsequently, linear regression was used to predict brain cortical activity during exercise and maximal power from resting EEG. The adjusted Pearson's multivariate coefficient of determination and *β* as standardized coefficient are provided. For all statistical analyses, an alpha level of *p* ≤ 0.05 was accepted as significant.

## 3. Results

Brain cortical activity during exercise was recorded at 100% of the individual anaerobic threshold, which was 3.31 ± 0.37 W·kg^−1^ and 2.74 ± 0.45 W·kg^−1^ in HIGH and LOW (*F*(1,27) = 13.66; *p* = 0.001; eta^2^ = 0.336), respectively. Subjects with higher maximal oxygen consumption also reached greater maximal power (4.67 ± 0.33 W·kg^−1^ versus 4.11 ± 0.49 W·kg^−1^; *F*(1,27) = 13.28; *p* = 0.001; eta^2^ = 0.330) than the LOW group.

**Figure 1:**
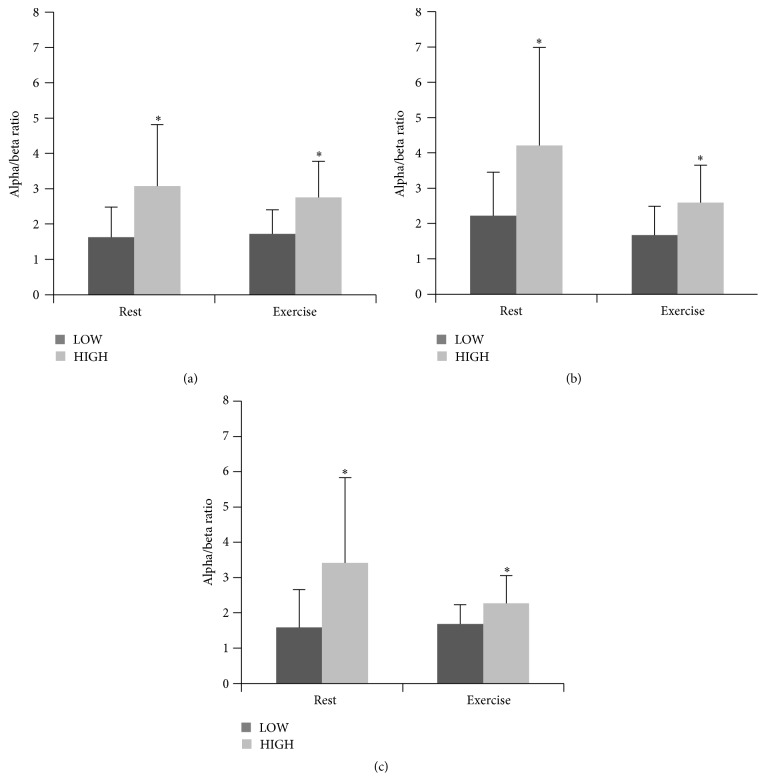
Alpha/beta ratio in HIGH and LOW at frontal (a), central (b), and parietal (c) electrode sites. ^*∗*^ *p* ≤ 0.05 compared to LOW.

The statistical analysis revealed main effects of group (*F*(1,27) = 12.04; *p* = 0.002; eta^2^ = 0.308), condition (*F*(1,27) = 4.24; *p* = 0.049; eta^2^ = 0.136), and region (*F*(2,26) = 8.50; *p* = 0.002; eta^2^ = 0.239) on subjects' alpha/beta ratio. Moreover, there was an interaction of condition and region (*F*(2,26) = 4.37; *p* = 0.025; eta^2^ = 0.139). Whereas alpha/beta ratio was increased at central brain region compared to parietal (*T*(28) = 3.36; *p* = 0.002) and frontal sites (*T*(28) = −2.74; *p* = 0.010) in the exercise condition, no regional differences were confirmed for the resting state. Furthermore, alpha/beta ratio was significantly different between HIGH and LOW at all electrode sites (Figure 1) in both the resting state (frontal: *T*(28) = −2.86; *p* = 0.008; central: *T*(28) = −2.52; *p* = 0.018; parietal: *T*(28) = −2.67; *p* = 0.013) and the exercise condition (frontal: *T*(28) = −3.19; *p* = 0.004; central: *T*(28) = −2.62; *p* = 0.014; parietal: *T*(28) = −2.37; *p* = 0.025).

When frequency was included as additional factor, interactions with condition (*F*(1,27) = 11.37; *p* = 0.002; eta^2^ = 0.296) and group (*F*(1,27) = 8.43; *p* = 0.007; eta^2^ = 0.238) were confirmed. Descriptive values of alpha and beta power are displayed in Table 2. Compared to the resting state alpha (*T*(28) = −7.39; *p* < 0.001) and beta power (*T*(27) = −12.43; *p* < 0.001) were significantly increased across electrode sites during exercise. Moreover, HIGH showed less beta power than LOW (*T*(28) = 2.45; *p* = 0.020).

The relation between resting EEG rhythms, brain cortical activity during exercise, and maximal power is shown in Table 3. Whereas alpha/beta ratio at rest explained 57% of variance of alpha/beta ratio during exercise at frontal brain region, resting EEG at other electrode sites did not predict brain cortical activity during exercise. When frontal alpha/beta ratio at rest was used as the independent variable for the prediction of maximal power, the model was able to explain 15% of the variance compared to 18% and 26% for the use of central and parietal alpha/beta ratio, respectively.

## 4. Discussion

Compared to the resting state, the alpha/beta ratio was decreased during cycling exercise as beta power increased more than alpha power. This change towards a higher level of arousal or vigilance has also been reported in a recent review by Ludyga et al. [28]. Increased beta activity reflecting higher cortical activation might be the result of greater processing demands during exercise and the tendency of the sensorimotor system to maintain the current network set [17]. The decrease of the alpha/beta ratio from rest to exercise was most pronounced at central brain region, where inter alia sensorimotor information of lower extremities is integrated [29]. In this respect, the maintenance of pedaling movements is associated with increasing feedback from sensory afferents and feed-forward due to the recruitment of motor neurons [12, 30].

Although the magnitude of the changes in alpha/beta ratio from rest to exercise was similar between groups, HIGH compared to LOW had a higher index in both conditions. This indicates a lower level of arousal in cyclists with high maximal oxygen consumption. Differences between athletes of high and low training or performance levels have also been observed at rest previously [4, 21]. Babiloni et al. [15] found enhanced cortical synchronization of pyramidal neurons reflected by increased alpha activity in elite karate athletes. As alpha power serves as an inverse indicator of mental alertness or arousal [18, 19], this implies that athletes are more efficient in posing cortical neurons into a nonoperative mode. Consequently, it can be assumed that a lower level of arousal at rest, which was also observed in the present trial, is due to greater relaxation ability in subjects with higher maximal oxygen consumption. This is further supported by a lower beta power in HIGH, because a decrease in beta power has been reported to be the EEG response to relaxation [31]. By maintaining a low arousal at rest, attentional resources may be reserved for a subsequent recruitment in movement-related tasks and associated information processing. The present results support this assumption as high resting alpha/beta ratio was associated with high alpha/beta ratio at frontal brain region during exercise. In previous studies resting alpha and beta activity have also been related to different measures of cognitive and motor performance, so that neural efficiency in the resting state is suggested to predict task-related performance [13, 14]. This is also applicable to endurance exercise as arousal at rest was directly related to maximal power during cycling.

Similar to the resting state, cyclists in HIGH showed a higher alpha/beta ratio than LOW during exercise at the individual anaerobic threshold. They performed cycling with less cortical activity despite identical relative exercise intensity. This observation indicates that the neural efficiency hypothesis is applicable to endurance-trained athletes. The present findings are further supported by Ludyga et al. [12], who confirmed a negative correlation between changes of aerobic performance and cyclists' brain cortical activity after a 4-week training period. Other studies have also shown that training elicits a decrease of cortical activity [11] and the suppression of task-irrelevant cognitive processes [32, 33] during different movement-related tasks. Evidence from animal studies suggests that improved neural processing and increasing influence of the cerebellar–thalamic–cortical circuit are underlying mechanisms of training-induced enhancement of neural efficiency [34]. In this respect, Taniwaki et al. [35] have proposed that this increased influence reflects a change from internal to increasingly external and automatic processing of motor tasks. The execution of highly automated movements, such as handwriting, does not require much attentional resources [36]. Therefore, the lower level of arousal during exercise in HIGH could be explained by cycling becoming an increasingly automated process with training. The reservation of cortical sources in experts is further supported by the results of Hüttermann and Memmert [37], who showed that athletes compared to nonathletes had higher cognitive performance during cycling exercise of similar relative intensity.

Furthermore, improvements in circulatory capacity and cerebrovasculature have also been suggested as underlying mechanisms for differences in EEG spectral power between athletes and nonathletes [21, 38]. Maximal oxygen consumption is correlated with cerebral blood flow, which is considered as a proxy for assessing neurogenesis [39]. This indicates an increased formation rate of new neurons and improved cerebral blood flow in HIGH compared to LOW. These improvements elicited by endurance exercise are thought to improve energy supply by creating a more supportive and nutritive environment for the surrounding neurons [38]. However, future research is necessary to investigate whether or not an optimized energy supply accounts for enhanced neural efficiency during exercise.

A methodological concern of EEG recordings during exercise is the potential impact of physiologic and mechanical disturbances. To reduce the possibility and impact of artefacts, the present study used a data acquisition and processing approach, which has proven reliable [27]. Additionally, only experienced cyclists were included in the study, because their ability to stabilize the upper body at moderate to high intensity is necessary to reduce movement-related artefacts when EEG is recorded during exercise on a stationary bike. Furthermore, brain cortical activity during exercise was averaged over 3 measuring time points. Therefore, it remains unclear whether or not the development of the alpha/beta ratio over time was different between groups. Nonetheless, the present results indicate a higher level of arousal in cyclists with lower aerobic fitness. As this was observed in both conditions, it cannot be ruled out that differences in alpha/beta ratio during exercise between HIGH and LOW are mainly due to the differences observed in the resting state. Whereas previous studies also reported differences in alpha activity at rest between elite-athletes and nonathletes [15, 16, 40], this is not supported by the present findings. This could be due to a lack of high differences in performance and/or training levels between groups and the low sample size of the present study. However, the differences in alpha/beta ratio confirmed between groups imply that this index is sensitive to small differences in aerobic power. Nonetheless, further investigations with higher sample size and more aerobic fitness subgroups are warranted to test and extend the current findings. Furthermore, it remains unclear whether or not other variables related to neural efficiency, such as cognitive performance and intelligence [41], have influenced the observed differences between HIGH and LOW.

In conclusion, brain cortical activity measured with EEG is influenced by subjects' maximal oxygen consumption. Cyclists with high aerobic fitness compared to peers with less aerobic fitness are able to complete submaximal cycling exercise with a lower level of brain cortical activity. This indicates enhanced neural efficiency in subjects with high aerobic power, possibly due to the inhibition of task-irrelevant cognitive processes. Moreover the use of resting EEG rhythms as predictor for task performance is also applicable to cycling as resting state arousal predicted maximal power.

## Figures and Tables

**Table 1:** Anthropometric data, maximal oxygen consumption, and maximal power of HIGH and LOW.

	LOW (*n* = 6 f/9 m)		HIGH (*n* = 5 f/9 m)		*p*
	Mean	SD	Mean	SD	
Age [y]	27.1	3.7	27.4	2.8	0.856
Height [cm]	176.5	8.7	179.9	4.1	0.187
Body mass [kg]	68.4	9.9	76.8	7.5	0.016
VO_2MAX_ [mL·min^−1^·kg^−1^]	46.4	4.1	55.6	2.8	<0.001
*P*_MAX_ [W·kg^−1^]	4.11	0.49	4.67	0.33	0.001

**Table 2:** Alpha and beta power at rest and during cycling in LOW and HIGH.

	Frequency	Condition	LOW (*n* = 6 f/9 m)		HIGH (*n* = 5 f/9 m)	
			Mean	SD	Mean	SD
Frontal	Alpha power (*μ*V^2^)	Rest	0.047	0.016	0.057	0.023
		Exercise	0.087	0.041	0.120	0.073
	Beta power (*μ*V^2^)	Rest	0.036	0.020	0.021	0.008
		Exercise	0.056	0.028	0.042	0.016

Central	Alpha power (*μ*V^2^)	Rest	0.036	0.017	0.057	0.037
		Exercise	0.072	0.032	0.099	0.052
	Beta power (*μ*V^2^)	Rest	0.017	0.005	0.014	0.004
		Exercise	0.046	0.016	0.040	0.019

Parietal	Alpha power (*μ*V^2^)	Rest	0.059	0.027	0.085	0.070
		Exercise	0.118	0.046	0.128	0.085
	Beta power (*μ*V^2^)	Rest	0.042	0.018	0.026	0.008
		Exercise	0.073	0.029	0.055	0.023

**Table 3:** Prediction of alpha/beta ratio during exercise and maximal power by resting alpha/beta ratio in cyclists (*n* = 29).

Dependent variable	Predictor variable	Beta	*r* ^2^	Adjusted *r* ^2^	SE	*F*	*p*
*α*/*β* during exercise	Frontal *α*/*β* at rest	0.766	0.587	0.572	0.651	38.43	0.000
	Central *α*/*β* at rest	0.220	0.049	0.013	1.027	1.38	0.251
	Parietal *α*/*β* at rest	0.241	0.058	0.023	0.721	1.66	0.209

Maximal power	Frontal *α*/*β* at rest	0.426	0.181	0.151	0.462	5.98	0.021
	Central *α*/*β* at rest	0.455	0.207	0.178	0.454	7.07	0.013
	Parietal *α*/*β* at rest	0.506	0.256	0.256	0.440	9.27	0.005
